# Identification of Genes Involved in *Pseudomonas aeruginosa* Biofilm-Specific Resistance to Antibiotics

**DOI:** 10.1371/journal.pone.0061625

**Published:** 2013-04-24

**Authors:** Li Zhang, Meredith Fritsch, Lisa Hammond, Ryan Landreville, Cristina Slatculescu, Antonio Colavita, Thien-Fah Mah

**Affiliations:** 1 Department of Biochemistry, Microbiology and Immunology, University of Ottawa, Ottawa, Canada; 2 Ottawa Hospital Research Institute, Ottawa, Canada; 3 Dartmouth College, Hanover, New Hampshire, United States of America; Kansas State University, United States of America

## Abstract

*Pseudomonas aeruginosa* is a key opportunistic pathogen characterized by its biofilm formation ability and high-level multiple antibiotic resistance. By screening a library of random transposon insertion mutants with an increased biofilm-specifc antibiotic susceptibility, we previously identified 3 genes or operons of *P. aeruginosa* UCBPP-PA14 (*ndvB*, PA1875–1877 and *tssC1*) that do not affect biofilm formation but are involved in biofilm-specific antibiotic resistance. In this study, we demonstrate that PA0756–0757 (encoding a putative two-component regulatory system), PA2070 and PA5033 (encoding hypothetical proteins of unknown function) display increased expression in biofilm cells and also have a role in biofilm-specific antibiotic resistance. Furthermore, deletion of each of PA0756, PA2070 and PA5033 resulted in a significant reduction of lethality in *Caenorhabditis elegans*, indicating a role for these genes in both biofilm-specific antibiotic resistance and persistence *in vivo*. Together, these data suggest that these genes are potential targets for antimicrobial agents.

## Introduction

*Pseudomonas aeruginosa* is a well-established opportunistic pathogen capable of infecting animals and plants as demonstrated with strain UCBPP-PA14 (PA14) [1]. Gene expression differs in biofilm and planktonic cells and indeed, about 1% of *P. aeruginosa* genes including those related to antibiotic resistance and virulence factors show differential expression in the two growth modes [2]. Antibiotic resistance in biofilms is due to a combination of different factors that act together to result in a level of resistance that is greater than that of planktonic cultures [3]–[6]. These factors include slow penetration of antibiotic through the biofilm [7], persister formation [8] and the induction of a lipid modification operon by extracellular matrix DNA [9]. Over the past several years, we have described other novel antibiotic resistance mechanisms (e.g., glucan-mediated sequestration and the expression of a biofilm-specific efflux pump) that are only expressed in a biofilm [10]–[12].

In order to identify novel mechanisms responsible for biofilm-specific antibiotic resistance, we developed a high-throughput system to identify mutants of *P. aeruginosa* that do not develop the characteristic increase in resistance to antibiotics when grown in a biofilm [10]. Several mutants were identified in this screen. We have previously published descriptions of the analysis of 3 of these mutants involving, respectively, *ndvB*, PA1875–1877 and *tssC1* genes [10]–[13]. Analysis of deletion mutants of these genes revealed that these mutants share a common phenotype: they form biofilms and grow as well as the wild type strain, yet they display a significant decrease in biofilm-specific resistance relative to the wild type strain [10]–[12]. Furthermore, these mutants have no defect in planktonic antibiotic resistance. *ndvB* encodes a glucosyltransferase that is important for the synthesis of cyclic-β 1, 3 glucans [14]. We determined that *P. aeruginosa* cyclic glucans contribute to antibiotic resistance in biofilms by interacting with antibiotics and sequestering them away from their cellular targets [10]. *ndvB* is also important for expression of ethanol oxidation genes, suggesting multiple roles of NdvB [13]. PA1875–1877 is part of an operon that encodes an efflux pump that removes antibiotics from the cells within the biofilm [11]. *tssC1* is a component of a *P. aeruginosa* type VI secretion system that is involved in biofilm-specific antibiotic resistance through an unknown mechanism [12].

The three initially characterized biofilm-specific antibiotic resistance loci are not linked by cellular function, suggesting that there are multiple pathways leading to resistance in biofilms. To further support this hypothesis, the three novel genetic loci that are the subject of the current publication, PA0756–0757, PA2070 and PA5033, are not predicted to belong to similar pathways/functions. PA0756 and PA0757 are predicted to encode a two-component regulatory system. PA2070 and PA5033 are both predicted to encode hypothetical proteins of unknown function.

In this study, we investigate the roles of PA0756–0757, PA2070 and PA5033 in biofilm-specific antibiotic resistance. We also explore the impact of the inactivation of these genes in an *in vivo* pathogenesis model involving the nematode *Caenorhabditis elegans*.

## Materials and Methods

### Bacterial strains, media, and reagents

All *P. aeruginosa* strains used in this study were derivatives of the PA14 wild-type strain and are listed in Table 1. The Δ*ndvB*, Δ*tssC1* and ΔPA1874-1877 mutants have previously been reported elsewhere [10]–[12]. Additional mutants with unmarked deletion of PA0756–0757 (i.e., PA14_54510-54500), PA2070 (PA14_37730) or PA5033 (PA14_66540) gene were constructed in PA14 by allelic exchange with pEX18Gm derivatives as previously described [10], [15]. Genes were cloned into the pJB866 vector as previously described [11], with open reading frames inserted downstream from the P*m* promoter, which is capable of induction by *m-*toluic acid [16]. Primers used for the construction of deletion mutants and pJB866 derivatives are listed in Table 2. Bacterial cells were grown at 37°C in rich medium (Luria-Bertani [LB] broth) or minimal medium. The minimal medium (M63-arginine) contained M63 salts (1% potassium phosphate [monobasic] and 0.2% ammonium sulfate) supplemented with arginine (0.4%) and MgSO_4_ (1 mM) [17]. Tobramycin, gentamicin, and ciprofloxacin were purchased from Research Production International (Mt. Prospect, Illinois, USA), Sigma-Aldrich Canada Ltd (Oakville, Ontario, Canada) and MP Biomedicals (Solon, Ohio, USA), respectively.

**Table 1:** *P. aeruginosa* strains and plasmids used in this study.

Strain or plasmid	Description	Source
Strain		
PA14	*P. aeruginosa* burn wound isolate	[1]
TFM15	PA14 Δ*ndvB*	[10]
TFM49	PA14 ΔPA2070	This study
TFM50	PA14 Δ*tssC1*	[12]
TFM57	PA14 ΔPA5033	This study
TMF65	PA14 ΔPA1874-1877	[11]
TFM73	PA14 ΔPA0756-0757	This study
TFM158	PA14 Δ*ndvB* ΔPA0756-0757	This study
Plasmid		
pEX18Gm	Broad-host-range gene replacement vector, *sacB*, gentamicin resistance	[15]
pJB866	Expression vector containing the *m*-toluic acid inducible promoter P*m*; tetracycline resistance	[16]
pJB866-PA0756-0757	pJB866 with the PA0756-0757genes inserted downstream from the *Pm* promoter	This study
pJB866-PA2070	pJB866 with the PA2070 gene inserted downstream from the *Pm* promoter	This study
pJB866-PA5033	pJB866 with the PA5033gene inserted downstream from the *Pm* promoter	This study

**Table 2:** Primers used in this study.

Primer	Sequence (5′ to 3′)*	Function
PA0756 F1	CACT**GAATTC**TGCCAAGGCTGTAGGTGAAC	Deletion of *PA0756-0757*
PA0756 R1	GTTC**TCTAGA**CGAGCTGCGGATGATCTTCCA	Deletion of *PA0756-0757*
PA0757 F2	GTTG**TCTAGA**TTCCCGCCGCCGACCGCGAGC	Deletion of *PA0756-0757*
PA0757 R2	TGAGC**AAGCTT**GCCGACAAAGTCCAACAGG	Deletion of *PA0756-0757*
PA2070 F1	ACTG**GAGCTC**ATGGCGCTCGTCGTGCTTCT	Deletion of *PA2070*
PA2070 R1	GTTA**TCTAGA**GCCGGTCACCTCGATTCGTT	Deletion of *PA2070*
PA2070 F2	GTTG**TCTAGA**TTCAGCAACCAGACCTACAC	Deletion of *PA2070*
PA2070 R2	AGTC**AAGCTT**ACCAGCAGGTCTTCGGAGTG	Deletion of *PA2070*
PA5033 F1	AGCA**GAATTC**ACCATCACCGCTGCCTATCC	Deletion of *PA5033*
PA5033 R1	TTAA**TCTAGA**ACCACGTTGCCGAAGCTGT	Deletion of *PA5033*
PA5033 F2	TTAA**TCTAGA**ATACAGCATCGGCGCCAGC	Deletion of *PA5033*
PA5033 R2	GTGT**AAGCTT**CCGCAAGGTGGTCTCGTC	Deletion of *PA5033*
PA0756 JB-F	GTTG**GAGCTC**ATGCGCATCCTTCTGGTGGAA	Cloning *PA0756-0757*into pJB866
PA0757 JB-R	GTTGAAGCTTGGAAGCCGCCACCAGCATGCTG	Cloning *PA0756-0757*into pJB866
PA2070 F7	CAAC**GAGCTC**ATGCACCGATCGCTCCAC	Cloning *PA2070* into pJB866
PA2070 R7	GTTG**AAGCTT**GCGCCGGCATCAGAAGCTGT	Cloning *PA2070* into pJB866
PA5033 F7	CAACGAGCTCATGAAACCAGCGATCAAGCG	Cloning *PA5033*into pJB866
PA5033 R7	GTTCAAGCTTCCTCCGGCGCGTTGGTTGGC	Cloning *PA5033*into pJB866
rpoD F3	CATCCGCATGATCAACGACA	qPCR
rpoD R2	GATCGATATAGCCGCTGAGG	qPCR
PA0756 F4	TCGGTGGCGAACAGTTGCAG	qPCR
PA0756 R4	GGCCAGTTGCTCCTTGCTCA	qPCR
PA2070 F4	CTCCGCGGTGGATCTCAACA	qPCR
PA2070 R4	GTCGAAGCGGCCTTCGTTCA	qPCR
PA5033 F4	GGCGTTCTGGTAGGAACCTG	qPCR
PA5033 R4	AGACCACGTTGCCGAAGCTG	qPCR

* Locations of restriction sites are underlined and in bold.

### Biofilm formation assays

These were conducted by using both the air liquid interface assay and the 96-well microtitre plate assay as described previously using M63-arginine medium [18]. Briefly, in the air liquid interface assay, appropriately diluted overnight cultures were added into the wells of the angled (30°) 6-well plates, followed by the incubation at 37°C for 24 h. After removing the culture from the wells and gently washing, the medium was added to cover the bottom of the wells and the biofilms were analyzed on an inverted microscope by phase-contrast microscopy. To quantify biofilm formation, biofilms were formed in 96-well microtitre plates for 32 h at 37°C, stained with 0.1% crystal violet, followed by the solubilization of the biofilm-associated crystal violet in 95% ethanol and the measurement of their optical densities at 550 nm [18]. The data obtained from two separate experiments (each with quadruplicate samples) were assessed by one-way analysis of variance (ANOVA).

### Quantitative real-time PCR (qPCR) analysis

RNA was extracted from planktonic cultures or colony biofilms grown at 37°C. Planktonic cultures were grown in M63-arginine medium to exponential phase. Colony biofilms on M63-arginine agar plates were prepared by distributing single drops of a saturated overnight culture of each strain on the surface of the agar plate (one strain per plate). The plates were incubated for 24 h at 37°C followed by 16 h at room temperature [11]. Colonies were scraped from the surface of the plates and RNA extraction and cDNA synthesis were performed as previously described [11]. cDNA was quantified using SYBR-green detection of PCR products with the MyiQ single-color detection system (Bio-Rad). Each 20 µl qPCR reaction contained 2 µl cDNA (∼1.2 µg), 10 µl SYBR Green PCR Master Mix (Applied BioSystems), and 100 pmol of each primer. The following thermal cycler conditions were used: 90 s at 95°C, followed by 45 cycles (with one cycle consisting of 60 s at 95°C, 30 s at 56°C, and 30 s at 72°C). qPCR primers are listed in Table 2. RNA isolated from at least two independent cell cultures were each tested in triplicate qPCR reactions, with expression of *rpoD* used as a reference standard. Statistical significance was determined by Student's *t*-tests.

### Antibiotic resistance assays

Minimum bactericidal concentrations were determined for biofilm (MBC-B) and planktonic (MBC-P) cultures as previously described [10], [11]. Briefly, overnight cultures of bacteria were diluted (1∶50) in M63-arginine in 96-well microtitre plates. For MBC-B assays, biofilms were formed over 24 h, planktonic cells were removed, and the biofilms were exposed to serial dilutions of antibiotics (ranged from 6.25 to 400 µg/ml for tobramycin, 12.5 to 800 µg/ml for gentamicin, and 2.5 to 160 µg/ml for ciprofloxacin). For MBC-P assays, antibiotics were added at the same time the plates were inoculated with the following concentrations used: 1 to 64 µg/ml for tobramycin, 2 to 128 µg/ml for gentamicin, and 0.5 to 32 µg/ml for ciprofloxacin. After 24 h of antibiotic treatment, bacterial survival was determined by spotting a small amount (ca. 3 µl) of culture on LB agar plates immediately after antibiotic treatment (MBC-P) or following a 24 h recovery period in which surviving cells can detach from biofilms into antibiotic-free growth media (MBC-B).

For another measure of planktonic antibiotic resistance, minimum inhibitory concentrations (MICs) were determined in LB broth using the two-fold broth dilution method [19]. For strains carrying pJB866 derivatives (Table 1), MICs were determined in LB or M63-arginine containing 2 mM *m*-toluic acid to induce expression of cloned genes. The concentrations of antibiotics ranged from 0.0625 to 64 µg/ml.

### *C. elegans* slow-killing assay

The *C. elegans* N2 Bristol wild-type strain was maintained using nematode growth medium and the *P. aeruginosa* slow-killing assay was conducted as described [20]. Briefly, *P. aeruginosa* strains were seeded onto slow killing low osmolarity plates. These plates were incubated at 37°C for 24 hours and then room temperature for 24 hours. Thirty synchronized L4 or young adult worms were placed onto the slow killing plates and the plates were incubated at 25°C for a total of 72 hours. Viability of the worms was assessed every 24 hours by gently stroking them. Worms that did not move were scored as dead and were removed from the plate. *Escherichia coli* OP50 is a standard food source of *C. elegans* and served as a control in this experiment.

## Results

### Characterization of three novel biofilm-specific antibiotic resistance genes

A genetic screen was previously conducted in order to identify novel genes involved in biofilm-specific resistance to the antibiotic tobramycin in *P. aeruginosa* PA14 [10]. Several candidate transposon-insertion mutants with increased biofilm-specific susceptibility to tobramycin were isolated from the initial screen. After conducting secondary screening (growth and biofilm formation; data not shown), six mutants that could form biofilms and grow as well as the wild-type strain, were selected for further study. The transposon element carried by each of the mutant strains was cloned and the DNA sequence flanking the transposon was determined and compared to the completed genome of *P. aeruginosa* [21]. We have completed the initial characterization of three genes/operon, *ndvB*, PA1875-1877 and *tssC1* [10]–[13]. In the current study, we focus on PA0756-0757 (an operon encoding regulator and sensor histidine kinase of a putative two-component regulatory system), PA2070 (encoding a hypothetical membrane protein), and PA5033 (encoding a hypothetical protein).

**Figure 1:**
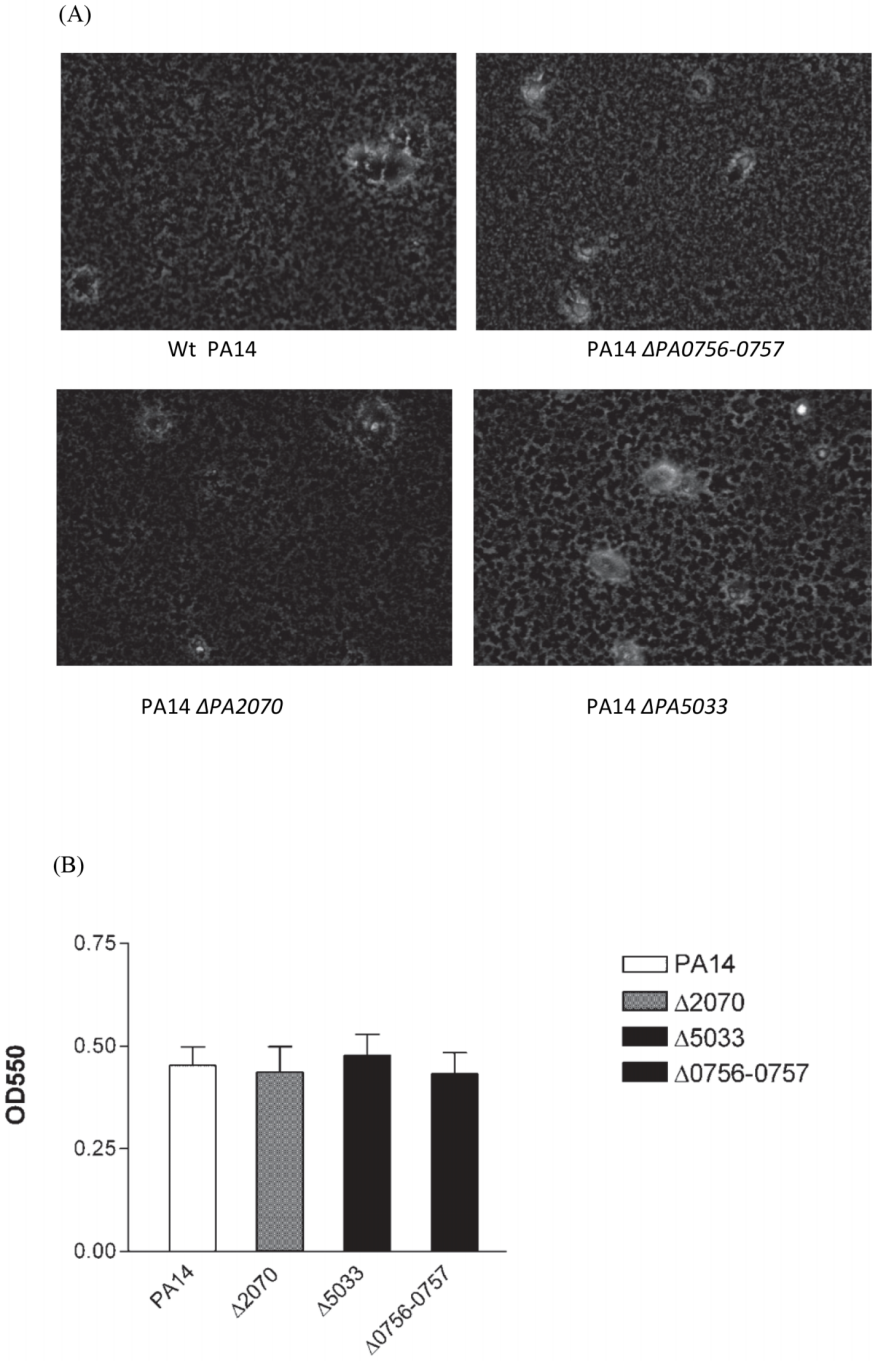
Inactivation of PA0756-0757, PA2070 or PA5033 does not affect biofilm formation. A. Air-liquid interface assay of biofilm formation of *P. aeruginosa* PA14 wild type and its deletion mutants, ΔPA0765-0757, ΔPA2070 and ΔPA5033. The images of biofilms at 200 X magnification were taken by phase-contrast microscopy after 24 h of growth at 37°C in M63-arginine medium. B. Quantification of biofilm formation by measurement of the biofilm-associated crystal violet (OD_550_). The data shown are the mean ± standard deviation from two separate experiments (each with quadruplicate samples) using the microtitre plate biofilm assay and display no significant difference (*p*>0.05) among the four groups as determined by one-way ANOVA.

In order to assess the importance of PA0756-0757, PA2070 and PA5033 in biofilm-specific antibiotic resistance, we generated in-frame deletions of PA2070 and PA5033 as well as the entire PA0756-0757 locus. The resulting deletion mutants, ΔPA0765-0757, ΔPA2070 and ΔPA5033, were assessed for their growth, biofilm formation and antibiotic resistance phenotype. All of these mutants exhibited a wild-type level of growth in both LB and M63 medium (data not shown). They were also competent in biofilm formation as shown by representative images of biofilms that were formed in 6-well microtitre dishes in the air liquid interface assay (Figure 1A) as well as by the quantification of the biofilm formation from the microtitre plate assay (Figure 1B). The antibiotic resistance phenotype obtained for these mutants by performing MBC-P and MBC-B assays are shown in Table 3. The previously characterized Δ*ndvB* mutant was included as control [10]. Three typical anti-pseudomonal antibiotics, tobramycin, gentamicin and ciprofloxacin, were tested. The MBC-P and MBC-B assays are static assays performed in sterile 96-well microtitre plates. The MBC-P assay is a measure of planktonic resistance that can be directly compared to MBC-B values that are indicative of resistance within a biofilm [10]. All three deletion mutants displayed reduced biofilm-specific resistance to tobramycin and gentamicin while showing no defect in resistance to ciprofloxacin. In the MBC-P assay, the ΔPA5033 mutant was more sensitive to tobramycin and gentamicin, the ΔPA0765-0757 mutant was also more sensitive to tobramycin, while the sensitivity of the ΔPA2070 mutant to three antibiotics tested was unaffected (Table 3).

**Table 3:** Minimal bactericidal concentrations of antibiotics for PA14 deletion mutants tested with planktonic cells (MBC-P) and biofilm cells (MBC-B) (µg/ml)^a^.

Strain	Tobramycin		Gentamicin		Ciprofloxacin	
	MBC-P	MBC-B	MBC-P	MBC-B	MBC-P	MBC-B
PA14	32	200	64	200–400	4	80
Δ*ndvB*	32	50	64	50–100	4	40
ΔPA2070	32	50	64	100	4	80
ΔPA5033	16	100	32	100–200	2–4	80
ΔPA0756-0757	16	25–50	64	100	2	80
Δ*ndvB* ΔPA0756-0757	16–32	12.5–25	64	25–50	2	40

a the values represent the mode of at least 6 biological replicates.

Since the ΔPA0756-0757 and ΔPA5033 deletion mutants displayed increased sensitivity to tobramycin or gentamicin in the MBC-P assays, we further assessed planktonic antibiotic resistance of the deletion mutants using the standard minimal inhibitory concentration (MIC) assay. ΔPA0756-0757, ΔPA2070 and ΔPA5033 exhibited no defects in this assay (Table 4), when tested against tobramycin and gentamicin. However, the ΔPA0756-0757 deletion mutant was slightly more sensitive to ciprofloxacin.

**Table 4:** Antibiotic susceptibility of PA14 deletion mutants assayed in LB broth.

Strain	Minimal inhibitory concentration (MIC; µg/ml)		
	Tobramycin	Gentamicin	Ciprofloxacin
PA14	2	2	0.5
Δ*ndvB*	2	2	0.5
ΔPA2070	2	1–2	0.5
ΔPA5033	2	2	0.5
ΔPA0756-0757	2	2	0.25
Δ*ndvB* ΔPA0756-0757	2	2	0.25

Taken together with the biofilm formation, MBC-P and MIC data showing that ΔPA0756-0757, ΔPA2070 and ΔPA5033 generally had no defect in these assays, we concluded that these deletion mutants were specifically deficient in antibiotic resistance only when growing in biofilms.

### PA0756-0757, PA2070 and PA5033 display a biofilm-specific expression pattern

**Figure 2:**
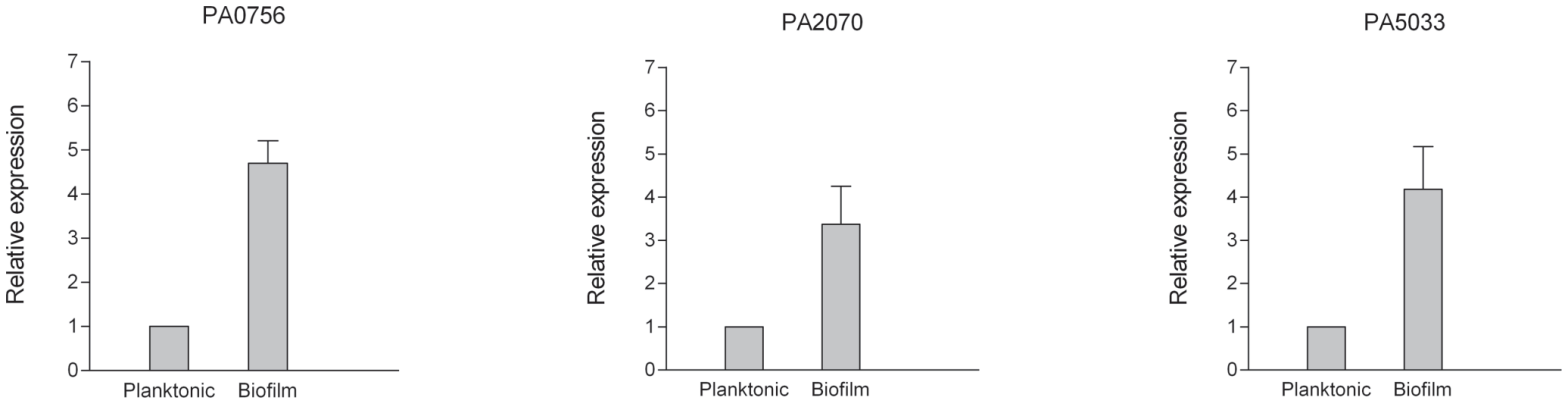
qPCR analysis of the expression of PA0756, PA2070 and PA5033 in planktonic and biofilm cells. For each condition, three biological replicate samples were tested in triplicate qPCRs.

The previously-characterized *ndvB*, PA1875-1877 and *tssC1* genes each exhibit a higher level of gene expression in biofilm cells, compared to planktonic cells [10]–[12]. In order to determine if PA0756-0757, PA2070 and PA5033 were similarly regulated, we performed quantitative mRNA-based real-time PCR experiments comparing the expression of these genes in planktonic- and biofilm-grown cells (Figure 2). All three loci exhibited a higher level of gene expression in biofilm cells, suggesting that these genes are important for biofilm resistance, but not planktonic resistance, because their expression is limited to biofilms.

### Cloned PA0756-0757, PA2070 and PA5033 genes increase the resistance of planktonic cells

In order to assess whether PA0756-0757, PA2070 and PA5033 can promote antibiotic resistance when overexpressed from a vector in planktonic cells, intact PA0756-0757, PA2070 and PA5033 genes from PA14 were individually cloned into a board-host-range vector, pJB866, which carries the *m*-toluic acid-controlled *Pm* promoter [16]. Compared with planktonic PA14 cells carrying the vector only, the cells containing the cloned genes in pJB866 (pre-induced with *m*-toluic acid) showed decreased susceptibility to tobramycin (2-fold MIC change from 4 to 8 µg/ml, gentamicin (4 to 8-fold MIC change from 2 to 8 or 16 µg/ml) and ciprofloxacin (2 to 4-fold MIC change from 0.25 to 0.5 or 1.0 µg/ml) (Table 5). These results suggest that PA0756-0757, PA2070 and PA5033 may act independently as antibiotic resistance genes. Taken together with the previously published data showing that *ndvB*, PA1875-1877 and *tssC1* are also able to increase planktonic antibiotic resistance when expressed from a vector in planktonic cells, this suggests that a mechanism of biofilm-specific expression likely contributes to biofilm-specific antibiotic resistance.

**Table 5:** Antibiotic susceptibility of PA14 deletion mutants containing pJB866-based plasmids and assayed in LB.

Strain	*m*-toluic acid induction (2 mM)	Minimal inhibitory concentration (MIC; µg/ml)		
		Tobramycin	Gentamicin	Ciprofloxacin
PA14 pJB866	−	2	2	0.25
	+	4	2	0.25
PA14 pJB866-PA2070	−	4	4	1
	+	8	16	1
PA14 pJB866-PA5033	−	4	4	0.25
	+	8	16	1
PA14 pJB866-PA0756-0757	−	2	4	0.25
	+	8	16	1

### *ndvB* and PA0756-0757 represent different biofilm-specific antibiotic resistance mechanisms

Previously, we showed that *ndvB* is important for biofilm-specific resistance to tobramycin through a mechanism involving sequestration of the antibiotic by *ndvB*-derived glucans [10]. To test whether *ndvB* and PA0756-0757 function in the same pathway, we constructed a deletion mutant combining the *ndvB* mutation and the PA0756-0757 deletions. The antibiotic resistance phenotype of the resulting mutant, Δ*ndvB*/ΔPA0756-0757, was determined in MBC-P and MBC-B assays (Table 3). In comparison to each single deletion mutant, the double mutant exhibited an increased sensitivity to tobramycin and gentamicin in the MBC-B assay, suggesting that the *ndvB*-mediated mechanism of antibiotic resistance is separate from the mechanism represented by PA0756-0757. This result is consistent with previous experiments with a Δ*ndvB*/ΔPA1874-1877 double mutant [11] and supports the theory that there are multiple mechanisms of resistance acting within a biofilm.

### Biofilm-specific antibiotic resistance genes contribute to PA14 persistence in a *C. elegans* slow-killing assay

**Figure 3:**
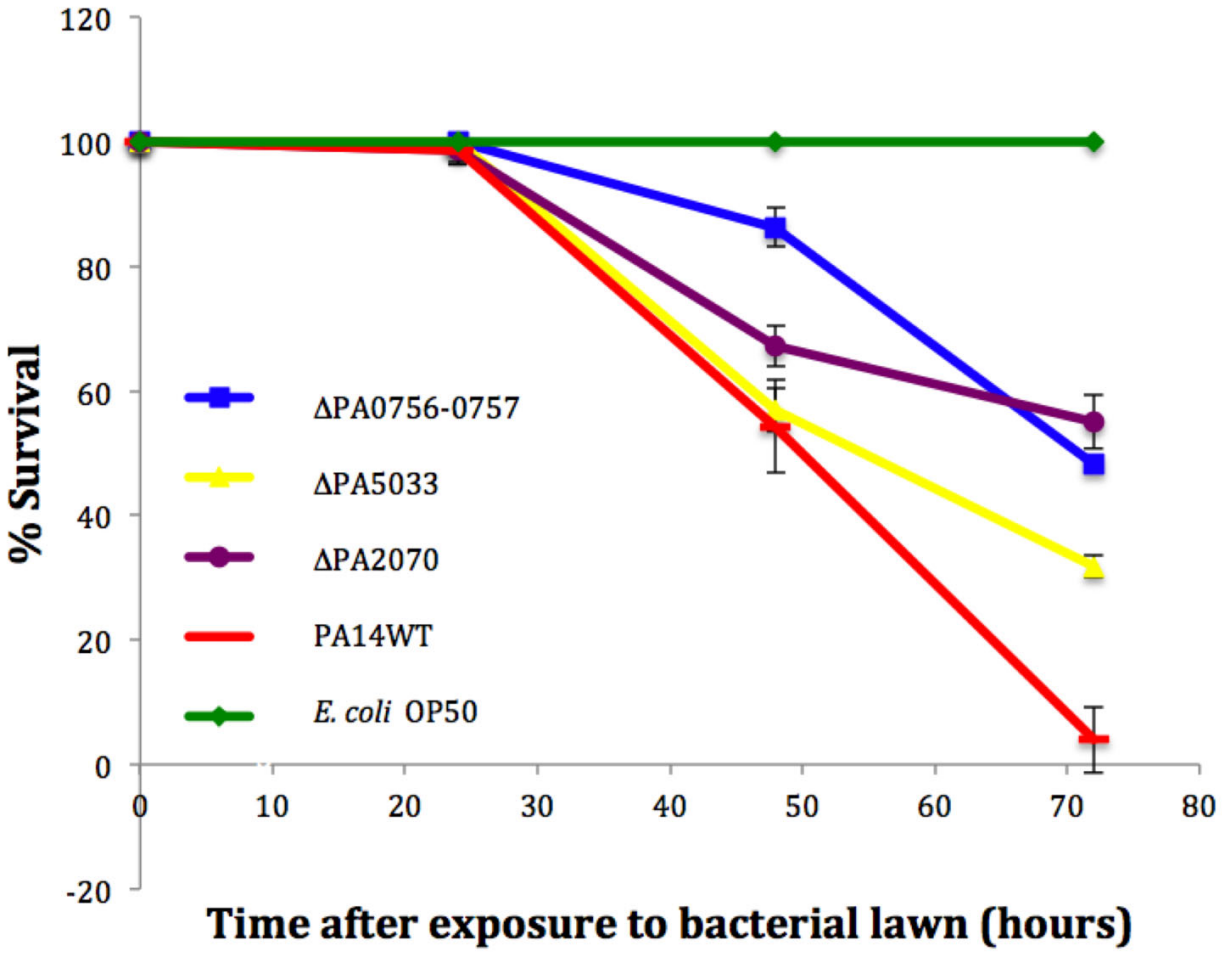
ΔPA0756-0757, ΔPA2070 and ΔPA5033 are attenuated in a *C. elegans* slow-killing model. Slow-killing conditions were used for each strain and death of *C. elegans* was measured every 24 h for a total of 72 h. Exposure to bacterial lawn represents when L4 or young adult hermaphrodite *C. elegans* were added to pathogenic plates. Values represent the results from at least three biological replicates. Error bars represent standard deviation, and lack of error bars means a standard deviation of zero.

*C. elegans* responds to pathogenic microbes including the lethal infections by *P. aeruginosa* and thus has been used as an *in vivo* pathogenesis model. *P. aeruginosa* can kill *C. elegans* in a slow-killing assay where death after the ingestion of the bacteria occurs within three days [22]. In contrast to the fast-killing assay that is dependent on the delivery of toxins to the host [23], the slow-killing assay is based on the ability of *P. aeruginosa* to accumulate in the intestine of the worm [22], [24], [25]. The exact mechanism of pathogenesis in the slow-killing assay is unclear but it is clearly an active process because heat-killed *P. aeruginosa* are not pathogenic in this model [22]. Since the slow-killing assay is described as an infectious process [26], it is likely that the initial steps of the infection entail the establishment of a biofilm and subsequent resistance to the host immune system. Thus, the slow-killing *C. elegans* assay measures the ability of *P. aeruginosa* to persist in the host, followed by active killing of the host. In order to address whether genes that are important for biofilm-specific antibiotic resistance *in vitro* are also important in a pathogenicity model that depends on *P. aeruginosa* persistence, we tested whether ΔPA0756-0757, ΔPA2070 and ΔPA5033 mutants were defective in the slow-killing *C. elegans* assay. The controls for the experiments were *P. aeruginosa* PA14 wild type as the positive control (0% survival at 72 hours) and *E. coli* OP50 as the negative control (100% survival at 72 hours). The three biofilm resistance gene deletion mutants were all attenuated in the slow-killing assay, with survival values ranging from 31.8% (ΔPA5033) to 55% (ΔPA2070) (Figure 3). Similar results were obtained when Δ*ndvB*, ΔPA1874-1877 and Δ*tssC1* were used in slow-killing *C. elegans* assay (Figure S1). An *sadC* mutant of *P. aeruginosa* defective in biofilm formation [27] was also included for comparison since biofilm formation-deficient mutants are attenuated in the *C. elegans* slow-killing assay, likely due to the fact that they do not accumulate in the worm intestine (Figure S1) [24]. These results suggest that there is a correlation between biofilm sensitivity to antibiotics *in vitro* and an inability to kill *C. elegans in vivo*. More *C. elegans* survived the exposure to the Δ*sadC* mutant (61.4%), suggesting that this mutant was cleared by the host more efficiently.

## Discussion

In this study, we have described the identification of three novel biofilm-specific antibiotic resistance genes in *P. aeruginosa*. Together with three previously characterized biofilm-specific antibiotic resistance genes, a total of six genes were identified in a genetic screen [10]–[12]. Each one of these genes is more highly expressed in biofilms, compared to planktonic cultures, and protects biofilm cells from antibiotics, likely through separate mechanisms. We also present evidence to suggest that *in vivo*, each gene is required to prevent the elimination of *P. aeruginosa* during an infection of *C. elegans* and therefore are important for persistence and pathogenesis *in vivo*.

PA0756 and PA0757 are predicted to encode a two-component regulatory system [21]. These types of systems relay information from outside of the cell into the cells and the result of this pathway is the response of the cells to a specific signal. In planktonic cultures, PA0756-0757 represses the expression of OpdH, a porin that is encoded by the upstream gene (PA0755) of PA0756-0757 operon transcribed in the opposite direction. OpdH is involved in tricarboxylate uptake [27]. However, it is not clear if this occurs in biofilms. Two genes immediately downstream of PA0756-0757 encode yet-uncharacterized hypothetical products. To date, several two-component regulatory systems have been implicated in biofilm formation, often as part of complex regulatory networks that govern the production of exopolysaccharides or the expression of surface appendages (reviewed in [28]), but this is the first two-component regulatory system to be identified to be important for biofilm-specific antibiotic resistance. The signal, or signals, that the PA0757 sensor histidine kinase responds to in biofilms has not been identified, although tricarboxylates are candidates. We predict that the downstream targets of the PA0756 response regulator will possibly be involved in biofilm-specific antibiotic resistance.

PA2070 and PA5033 are both predicted to encode hypothetical proteins of unknown function [21]. PA5033 has a type I export signal and the TIGRFAM predicts that PA2070 is a part of the TonB-dependent heme/hemoglobin receptor family. In fact, functional data has implicated PA2070 in a cell-surface signaling pathway that involves the extracytoplasmic function sigma factor PA2050 with possible function in metal uptake [29]. The genes upstream and downstream of PA2070 encode, respectively, elongation factor G (FusA2; PA2071) and a carbamoyl transferase (PA2069), while the gene products from upstream and downstream of PA5033 are, respectively, a putative transcriptional regulator (PA5032) and an uroporphyrinogen decarboxylase (HemE). None of these gene products are known to be involved in biofilms. Therefore, given their predicted locations/function, further investigation of the function of PA2070 and PA5033 proteins in biofilm-specific antibiotic resistance will likely provide insights on additional novel mechanisms of resistance.

The combination of mutations in *ndvB* and PA0756-0757 operon resulted in a mutant that was more sensitive to antibiotics compared to a single mutation in either genetic locus suggests that they represent separate resistance mechanisms. *ndvB* and PA1875-1877 also represent different resistance mechanisms [11]. Our current work suggests that there are additional mechanisms of resistance in biofilms, represented by PA0756-0757, PA2070 and PA5033, that have yet to be characterized. Taken together with the fact that other mechanisms, such as the induction of a lipid modification system [9], have been identified through different types of approaches, it is clear that biofilm-specific antibiotic resistance encompasses multiple mechanisms of resistance.

Loss of either *ndvB*, PA1874-1877, *tssC1*, PA0756-0757, PA2070 or PA5033 results in a deletion mutant that is more sensitive to antibiotics only when the bacteria are growing in a biofilm (Table 3) [10]–[12]. Despite the modest decreases in antibiotic resistance of each single deletion mutant *in vitro*, our results revealed that the 6 mutants tested are all deficient in the *in vivo C. elegans* slow killing assay. Depending on the exact mechanism of antibiotic resistance represented by each gene, the *in vitro* phenotype of antibiotic sensitivity could equate to *in vivo* sensitivity to host immune factors. A key signaling pathway in the *C. elegans* innate immune response is regulated by a conserved p38 MAP kinase [30], [31]. This pathway results in the expression of uncharacterized immune effectors that likely possess antimicrobial activities [31]. The mechanisms of resistance represented by *ndvB*, PA1874-1877, *tssC1*, PA0756-0757, PA2070 or PA5033 may be important for protection against antibiotics as well as the antimicrobial compounds produced by the *C. elegans* innate immune system.

The fast-killing *C. elegans* assay is a toxin-based mode of killing where the worms die within one day [22]–[24]. As such, it represents more of an acute type infection. In contrast, the slow-killing assay allows for the study of the host-pathogen interactions because microbes including *P. aeruginosa* can establish an infection within the intestine of the worm [25]. This infection is characterized by the accumulation of bacteria and a matrix in the intestine [25] and likely relies on the establishment of a biofilm within the intestine. The fact that the Δ*sadC*, a mutant that is impaired in biofilm formation, was more defective in the slow-killing assay compared to the biofilm antibiotic resistance gene deletion mutants, is consistent with the concept that biofilm formation is also an important aspect of *P. aeruginosa* persistence and pathogenesis in the *C. elegans* slow-killing model.

## Supporting Information

Figure S1 **Δ** ***ndvB*** **, ΔPA1874–1877, Δ** ***tssC1*** **and Δ** ***sadC*** **are attenuated in a** ***C. elegans*** **slow-killing model.** Slow-killing conditions were used for each strain and death of *C. elegans* was measured every 24 h for a total of 72 h. Exposure to bacterial lawn represents when L4 or young adult hermaphrodite *C. elegans* were added to pathogenic plates. Values represent the results from at least three biological replicates. Error bars represent standard deviation, and lack of error bars means a standard deviation of zero.
(TIFF)
