# Supplementary figures and images for: Identification of Genes Involved in Pseudomonas aeruginosa Biofilm-Specific Resistance to Antibiotics
Source: PLoS One. 2013 Apr 24;8(4):e61625. doi: 10.1371/journal.pone.0061625 (PMC3634840; doi:10.1371/journal.pone.0061625)

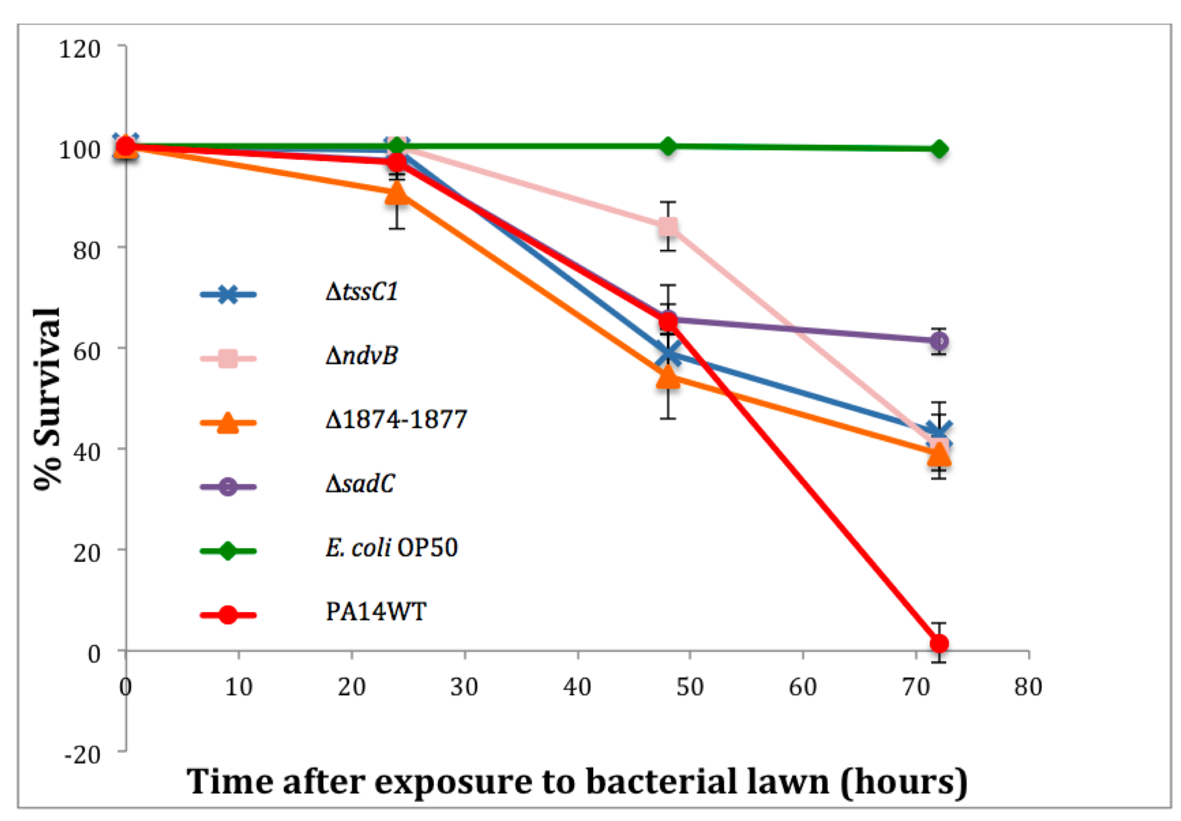

Supplement: Figure S1 — Δ ndvB , ΔPA1874–1877, Δ tssC1 and Δ sadC are attenuated in a C. elegans slow-killing model. Slow-killing conditions were used for each strain and death of C. elegans was measured every 24 h for a total of 72 h. Exposure to bacterial lawn represents when L4 or young adult hermaphrodite C. elegans were added to pathogenic plates. Values represent the results from at least three biological replicates. Error bars represent standard deviation, and lack of error bars means a standard deviation of zero. (TIFF) [file pone.0061625.s001.tif]
